# 存活10年以上非移植多发性骨髓瘤25例临床分析

**DOI:** 10.3760/cma.j.issn.0253-2727.2022.02.014

**Published:** 2022-02

**Authors:** 曼 申, 新 李, 光忠 杨, 佳佳 张, 然 汤, 仲夏 黄, 文明 陈

**Affiliations:** 首都医科大学附属北京朝阳医院血液科，北京 100020 Department of Hematology, Beijing Chao-Yang Hospital, Capital Medical University, Beijing 100020, China

多发性骨髓瘤（MM）是一种浆细胞恶性增殖性疾病，在肿瘤中占1％，在血液系统肿瘤中占10％ [1]。近年来随着新药陆续应用于临床，MM患者的缓解与生存得到极大改善，其中位生存期较传统化疗时代明显延长[2]，总生存期超过10年的患者被认为长期存活，其比例不足10％[3]–[4]。目前，一线接受造血干细胞移植被认为是MM患者长期存活的影响因素[5]–[6]，而有关非移植长期存活MM患者的临床特征报道少见。本研究回顾性分析我中心近年来收治的非移植MM患者，筛选出生存期达10年以上的病例并总结其临床特征。

## 病例与方法

1. 病例：本研究回顾性分析2005年7月至2011年7月我中心收治的301例非移植MM患者，生存期大于10年者共25例，在同期住院MM患者中占8.3％。

2. 治疗及疗效评估：25例患者的诱导化疗方案中，11例接受含硼替佐米的方案，包括BD方案（硼替佐米+地塞米松）和BCD方案（硼替佐米+环磷酰胺+地塞米松）；2例患者接受含沙利度胺的CTD方案（环磷酰胺+沙利度胺+地塞米松）；9例患者接受VAD方案（长春新碱+阿霉素+地塞米松）；3例患者接受M2方案（美法仑+卡莫司汀+环磷酰胺+长春新碱+泼尼松）。疗效判断参照国际骨髓瘤工作组（IMWG）的统一标准[7]，记录最佳疗效、最佳缓解时间（time to best response，TBR）、确诊1年时疗效、维持治疗方案及复发后再治疗疗效。深度缓解包括完全缓解（CR）及非常好的部分缓解（VGPR）。维持治疗定义为维持药物至少持续应用6个月。无进展生存（PFS）时间定义为自治疗之日起至疾病进展或随访终止时间，总生存（OS）时间定义为自确诊之日起至因任何原因死亡或随访终止时间。

3. 随访：通过查阅患者住院病历的方式随访，以死亡为随访终点，并记录患者的OS时间。随访至2021年7月，中位随访时间为136（120～193）个月。

4. 统计学处理：采用SPSS 24.0软件进行统计学分析。计量资料用*M*（范围）表示，计数资料用例数（百分比）表示。应用Kaplan-Meier法绘制患者OS及PFS曲线，生存曲线的比较采用Log-rank检验，*P*<0.05为差异有统计学意义。

## 结果

1. 临床特征：25例患者初诊时中位年龄为56（48～74）岁，65岁以上患者5例（20％）。男9例，女16例，ISS分期Ⅰ、Ⅱ期患者22例（88％），Ⅲ期患者3例（12％）。中位美国东部肿瘤协作组活动状态（ECOG-PS）评分1（1～2）分，≥2分患者5例（20％）。IgG型患者14例（56％），IgA型6例（24％），轻链型4例（16％），其他1例（4％）。MM轻链类型κ/λ＝1.0。肾功能不全患者1例（4％），高钙血症1例（4％），LDH升高1例（4％），HGB<100 g/L者7例（28％）。中位骨髓浆细胞比例17％（11％～84％），1例（4％）患者有髓外病变，无细胞遗传学高危患者。值得注意的是，10余年前细胞遗传学检测尚未普及，11例患者缺失初诊时细胞遗传学数据；14例中5例接受FISH检测，均为阴性，余9例进行了染色体G显带检测，其中8例为正常染色体核型，仅1例为非超二倍体复杂核型，根据IMWG的细胞遗传学危险分层，均为标危组[8]。

2. 治疗及疗效：本组患者初始诱导治疗后中位TBR为112（56～210）d，中位诱导治疗疗程数为4（3～8）个疗程，诱导治疗后14例（56％）患者获得CR，9例（36％）获得VGPR，2例（8％）患者获得部分缓解（PR），深度缓解率达92％。24例（96％）患者接受了维持治疗，4例（16％）患者接受干扰素、美法仑方案，20例（80％）接受免疫调节剂、硼替佐米方案。确诊1年时14例（56％）患者获得CR，深度缓解率为84％，总体反应率（ORR，达到PR及以上患者所占百分比）为100％。25例患者首次复发时有7例为症状复发，另外18例（72％）虽无症状但达到治疗指征即启动二线治疗。再治疗ORR为100％。本组患者中位治疗线数为4（2～6）线。

3. 生存：患者预计中位OS时间为148.0（138.0～157.9）个月，至随访结束，25例患者中有15例存活，其中2例（8％）患者已生存超过20年。25例患者诱导治疗后的中位PFS（PFS_1_）时间为58（17～111）个月，PFS_1_时间大于5年的患者有12例（48％）。根据诱导方案中是否含有硼替佐米和（或）沙利度胺分为新药及传统化疗两组，两组间PFS_1_时间的差异无统计学意义（58个月对50个月，*P*＝0.858）。首次复发后再治疗的中位PFS（PFS_2_）时间为28.5（4.0～132.0）个月，PFS_2_时间大于2年的患者有14例（56.0％），PFS_2_时间较PFS_1_时间明显缩短（28.5个月对58.0个月，*P*＝0.012）。

对10例死亡患者的死因进行分析，1例死于急性脑血管事件，余9例死于疾病进展，其中5例患者合并髓外病变，1例合并浆细胞白血病。值得注意的是，9例患者在多线（2～5线）治疗复发时合并髓外病变，7例出现骨旁髓外病变后中位生存21（4～125）个月，余2例患者出现非骨旁髓外病变后2个月内死亡（1例表现为胸腔积液浆细胞浸润，另1例为肺内浆细胞瘤）。合并第二肿瘤者2例，1例合并急性髓系白血病，1例合并结肠癌，均经病理证实。

## 讨论

MM是一种异质性较强的血液系统肿瘤，患者生存期差别较大。2020年版中国多发性骨髓瘤诊治指南中提出MM预后因素主要可以分为宿主因素、肿瘤特征、治疗方式及对治疗的反应三个方面[9]。本研究通过对我中心收治的MM患者进行长达10年的随访，筛选出25例生存期大于10年的非移植MM患者，观察其基线临床特征（宿主因素及肿瘤特征）及对治疗的反应。

基线临床特征方面，首先，本组患者中位确诊年龄为54岁，而文献报道的MM中位发病年龄为70岁[10]。一项纳入1 119例患者的研究中生存期大于10年MM患者的中位年龄为54岁[11]。另一项来自美国的研究证实，发病的中位年龄与生存期明确相关，年轻患者更易获得长期生存，可能与脏器功能好、更易耐受联合化疗有关[11]。其次，文献[11]，[13]报道HGB水平较高的患者预后良好，因其既能反映骨髓受累程度较轻，同时具有较好的化疗耐受性，本组有18例（72％）患者初诊HGB水平大于100 g/L。2015年IMWG在ISS分期的基础上引入细胞遗传学与LDH水平，提出了R-ISS分期系统，并报道R-ISS Ⅰ期、Ⅱ期、Ⅲ期患者的5年OS率分别为82％、62％、40％，5年PFS率分别为55％、36％、24％[14]。本组患者中ISS分期Ⅰ、Ⅱ期患者占88％，仅1例LDH升高，且5年PFS率为48％，与文献报道的R-ISS分期Ⅰ期患者相近，遗憾的是，本研究患者纳入时间至少为10年前，彼时细胞遗传学检测尚未普及，故无法进行R-ISS分期。

治疗反应方面，2018年Mellors等[15]指出，TBR是影响生存的重要因素之一，达到平台期至少需要120 d的患者的OS时间和PFS时间更长。TBR延长可能代表“持续应答者”表型，是一种独立于一线自体造血干细胞移植治疗、应答深度、ISS分期和细胞遗传学风险等生物标志物的生存优势[15]。此外，2019年一项前瞻性非随机临床试验纳入626例新诊断MM患者，其中早期反应者（TBR<90 d）的OS率较晚期反应者（TBR≥90 d）明显降低[16]。相似地，本组患者中位TBR为112 d，TBR>120 d者占44％，TBR>90 d者占92％，符合缓慢的缓解方式。

关于缓解深度与疗效维持两者在预示长期生存中孰轻孰重，不同学者存有争议[13],[17]。Usmani等[13]关注确诊1年时的CR率并指出患者维持CR状态1年对长期生存有益，该观点兼顾缓解深度及疗效维持两方面因素。Rodríguez-Otero等[18]认为MM病情控制达到5年预示长期生存。本研究的1年深度缓解率为84％，中位PFS_1_时间可达58个月，与上述结论相似，提示长期而稳定的深度缓解对OS期达到10年有帮助。

维持治疗有助于骨髓瘤残留灶的清除从而进一步延长PFS时间，此外维持治疗还能改善患者的生活质量[19]，本研究中96％患者接受过维持治疗。本组患者首次复发后新药（硼替佐米、免疫调节剂）的应用比例为80％，再治疗ORR可达100％，符合报道中提到的复发后应用新药可明显延长患者的OS时间[20]，再治疗有反应也可预示长期生存[11]。本研究的局限性为例数较少，未能进行统计学分析；时间跨度较长，治疗方案不统一；细胞遗传学检测率低等。
